# Supplementary material for: The ATF6β-calreticulin axis promotes neuronal survival under endoplasmic reticulum stress and excitotoxicity
Source: Sci Rep. 2021 Jun 22;11:13086. doi: 10.1038/s41598-021-92529-w (PMC8219835; doi:10.1038/s41598-021-92529-w)
Supplement: Supplementary file 1 — Supplementary Information. [file 41598_2021_92529_MOESM1_ESM.pdf]

The ATF6β-calreticulin axis promotes neuronal survival under endoplasmic reticulum stress and excitotoxicity

Dinh Thi Nguyen[1], Thuong Manh Le[1], Tsuyoshi Hattori[1], Mika Takarada-Iemata[1], Hiroshi Ishii[1], Jureepon Roboon[1], Takashi Tamatani[1], Takayuki Kannon[2], Kazuyoshi Hosomichi[2], Atsushi Tajima[2], Shusuke Taniuchi[3], Masato Miyake[3], Seiichi Oyadomari[3], Takashi Tanaka[4], Nobuo Kato[5], Shunsuke Saito, Kazutoshi Mori[4], Osamu Hori[1*]

Supplementary information

Supplementary tables (Table S1-S3)

Table S1 Differentially expressed genes in *Atf6b*$^{-/-}$ brain (p<0.05)

Downregulated genes in *Atf6b*$^{-/-}$ brain				
Gene	WT	*Atf6b*$^{-/-}$	log2 (fold-change)	p-value
Atf6b	31.633	7.66511	-2.04505	<5.00E-05
Rsph3a	11.5898	6.87324	-0.753794	0.03625
Slc4a1ap	14.1345	8.47099	-0.738618	0.00545
Calr	203.013	123.236	-0.720147	<5.00E-05
Tagap1	10.7218	7.09068	-0.596551	0.00155
Zfp46	16.4713	11.5521	-0.511808	0.0306
Ddx3y	12.4869	8.78594	-0.507145	0.00365
Thsd4	11.2284	8.18176	-0.456674	0.0046
Nkain1	10.2744	7.63411	-0.428519	0.027
Pla2g7	84.9424	64.1773	-0.404422	0.00865
Col6a1	33.9319	25.7853	-0.396092	0.01075
Calb2	41.538	31.5872	-0.395091	0.012
Fam107a	134.643	102.774	-0.389658	0.01485
Trpm3	10.1718	7.77531	-0.387606	0.01675
Sgk1	25.7941	19.7465	-0.385444	0.0236
Slc4a4	25.3338	19.5091	-0.376918	0.0152
Mt2	244.703	188.703	-0.374911	0.01655
Gpr17	13.7092	10.7602	-0.349437	0.0296
Mal	75.9335	59.7091	-0.346785	0.0185
Slit3	13.5417	10.8053	-0.325672	0.0341
Col25a1	19.4255	15.5616	-0.319957	0.04735
AC149090.1	300.785	242.572	-0.310319	0.04235

Upregulated genes in *Atf6b*$^{-/-}$ brain				
Gene	WT	*Atf6b*$^{-/-}$	log2 (fold-change)	p-value
Gm47283	13.354	43.6105	1.70741	<5.00E-05
Igf2	19.0054	39.9251	1.07089	<5.00E-05
Ptgds	452.8	942.637	1.05783	<5.00E-05
Mgp	20.9161	35.9567	0.781646	<5.00E-05
Hspa5	121.782	195.426	0.682327	0.00145
Igfbp2	16.5051	26.1894	0.666073	2.50E-04
Pacrg	15.6079	24.531	0.652331	0.0077
Fxyd5	14.7043	22.6957	0.626184	0.00605
Pdia4	16.5207	24.6939	0.579886	3.00E-04
Tmem181b-ps	50.8316	72.4126	0.510517	0.0072
H2-D1	12.1692	17.213	0.500263	0.0353
Neat1	34.358	48.3574	0.493091	0.0025
Ly6a	18.7354	26.0661	0.47641	0.0085
Cars	17.0994	23.7093	0.471512	0.04035
H2-K1	16.9189	23.4385	0.470241	0.03755
Myl9	11.9739	16.2991	0.444894	0.01375
Apod	85.8839	116.502	0.439901	0.0043
Psrc1	10.3895	13.8884	0.418754	0.03945
Bex1	34.0072	44.8394	0.398926	0.0186
Fxyd7	47.639	61.2804	0.363284	0.02985
Egr1	19.154	24.279	0.342066	0.0258
Dnajb11	24.9619	31.5182	0.336456	0.03615
Atf4	60.6921	76.4419	0.332855	0.0253
Wfs1	22.6167	28.3747	0.327215	0.04035
P4ha1	19.794	24.6951	0.319167	0.04445
Rgs7	23.7278	29.4115	0.309803	0.04605
Stmn1	146.103	179.343	0.295739	0.048

Table S2 Expression of genes encoding molecular chaperones in the ER in WT and *Atf6*$^{-/-}$ mouse brains

Gene	WT	*Atf6b*$^{-/-}$	log2 (fold-change)	p-value
Calr	203.013	123.236	-0.720147	<5.00E-05
Hspa5 (GRP78)	121.782	195.426	0.00145	0.00145
Hsp90b1 (GRP94)	110.637	128.981	0.1398	0.1398
Hyou1 (GRP170/ ORP150))	26.6409	27.9665	0.07006	0.66575
Pdia3 (ERp57)	71.9795	69.394	0.7614	0.7614
Pdia4 (ERp72)	16.5207	24.6939	0.0003	0.0003
Pdia6 *(ERp5)*	54.1244	67.2112	0.312422	0.0606
Pdia10 (ERp44)	15.0019	15.8719	0.0813263	0.7464
Hsp40 (Dnajb11)	24.9619	31.5182	0.336456	0.03615
Canx	103.499	102.51	-0.0138629	0.94155

Table S3 List of qRT-PCR primers used in this study

Gene (mouse)	Primer sequences (5'-3') Forward	Primer sequences (5'-3') Reverse
Atf6b	AACAGGAAGGTTGTCTGCATCAT	CTGCTCATCCGAGGAGAGACATG
Atf6a	ACAGCTGCCTAACCATGTG	AGCGATATCCGAACCCATAC
Calr	ATAAAGGGCTGCAGACAAGC	CCACAGTCGATATTCTGCTC
Canx	AGCAGAGATGGCATGATGCT	GTTGAGGCTCATGATGGACA
Hsp90b1(GRP94)	AAGGCCCTCAAGGACAAGAT	TGCCAGACCATCCATCCATACTGA
Hspa5 (GRP78)	ATGGTATTCTCCGAGTGACA	GCTTTCCAGCCATTCAATCT
Fos	CTGCAGCCAAGTGCCGGAATC	GGCAATCTCAGTCTGCAACGC
Fosb	TTTTCCCGGAGACTACGACTC	GTGATTGCGGTGACCGTTG
Bdnf	ACAAGGCAACTTGGCCTACC	AGCAGCTCTTCGATGACGTG

Supplementary figure legends

Figure S1. Expression of *Atf6a* and *Atf6b*. Expression of *Atf6a* (A, B, D) and *Atf6b* (C) mRNAs. Total RNA was isolated from the indicated samples and qRT-PCR was performed. HPC: hippocampus, Cx: cerebral cortex, SC: spinal cord. A, n=5 mice. B, n=4 experiments. C, n=4 (male) and n=3 (female) mice. D, n=4 experiments. Data are shown as mean ± SEM.

*p < 0.05, **p < 0.001, ***p < 0.001 by a one-way ANOVA followed by the Tukey test.

Figure S2. Read alignments of RNA-sequencing for *Atf6b*. RNA-sequencing read alignments from the hippocampus of WT (upper row) and *Atf6b*$^{-/-}$ (lower row) mice are illustrated by Integrated Genomics Viewer (http://software.broadinstitute.org/software/igv/). The snapshot shows an enlarged view for these alignments within the *Atf6b* region. The gray bar chart at the top of each alignment (Coverage track) shows the depth of reads at each locus, with the number of reads ranging from 0 to 500. The exons of *Atf6b* gene are indicated on the bottom.

Figure S3. Expression of molecular chaperones in the ER and promoter activities for CRT and GRP78. A, Expression of molecular chaperones in the ER in WT, *Atf6a*$^{-/-}$ and *Atf6b*$^{-/-}$ hippocampi. Total RNA was isolated from the CA3 region of the hippocampus and qRT-PCR was performed. n=5–6 mice. Data are shown as mean ± SEM. *p < 0.05, **p < 0.01, ***p < 0.001 by a one-way ANOVA followed by the Tukey test. B, C, Reporter assays using WT and *Atf6b*$^{-/-}$ MEFs. B, The CAT ELISA and luciferase assay were performed using cells transfected with the mouse CRT promoter pCC1 (left) and the human CRT promoter huCRT (right), respectively. C, The luciferase assay were performed using cells transfected with the human GRP78 promoter huGRP78, n=6. Data are shown as mean ± SEM. **p < 0.01, ***p < 0.001 between two genotypes and ##p < 0.01 compared to normal conditions by a two-way ANOVA followed by the Bonferroni test.

Figure S4. Expression of Ca^{2+}-indicator proteins in the primary hippocampal neurons. A, After transfection of cDNAs for each Ca^{2+}-indicator protein, cells were fixed and subjected to immunocytochemical staining with GFP antibody. n=50–60 cells in each condition from two independent experiments. Data are shown as mean ± SEM. Scale bar: 10 μm.

Figure S5. ER stress-induced death in the primary hippocampal neurons. Nuclear localization of EthD-1 in Figure 4A is shown by the images with single fluorescence for Hoechst 33342 and EthD-1. Scale bar: 10 μm.

Figure S6. Protection of Neuro2a cells by ATF6β. A, Gene silencing of *Atf6b* in Neuro 2a cells. Neuro 2a cells were transfected with ATF6β-siRNA1, ATF6β-siRNA2, or control-siRNA, and qRT-PCR was performed after 24 h. n=4 experiments. Data are shown as mean ± SEM. ***$p < 0.001$ by a one-way ANOVA followed by the Tukey test. B, C, LIVE/DEAD viability assay (B) and immunocytochemistry (C). Neuro 2a cells transfected with ATF6β-siRNA1, ATF6β-siRNA2, or control-siRNA were incubated for 48 h and treated with Tg (20 nM) or Tm (5 μg/ml). Cell survival/death and apoptosis were evaluated by the LIVE/DEAD viability assay at 36 h (B) and immunocytochemical staining for cleaved caspase-3 at 24 h after stimulation (C), respectively. n=3 in (B) and n=4 in (C). Data are shown as mean ± SEM. *$p < 0.05$, **$p < 0.01$, ***$p < 0.001$ by a two-way ANOVA followed by the Bonferroni test. Scale bar: 15 μm.

Figure S7. Protection of the primary hippocampal neurons by treatment with Ca^{2+}/ER

stress-modulating compounds. A, WT and *Atf6b*$^{-/-}$ hippocampal neurons were treated with Tm (1 μg/ml) together with BAPTA-AM (5 μM), 2-APB (2 μM), or salubrinal (5 μM). Cell death was evaluated by immunocytochemical staining for cleaved caspase-3. Typical images related to Figure 5C are shown. Scale bar: 20 μm. B, WT and *Atf6b*$^{-/-}$ hippocampal neurons were treated with BAPTA-AM (5 μM) or 2-APB (2 μM) alone. Cell death was evaluated by immunocytochemical staining for cleaved caspase-3. n=3 experiments. Data are shown as mean ± SEM. ***p< 0.001 by a two-way ANOVA followed by the Bonferroni test. Scale bar: 20 μm. C, WT and *Atf6b*$^{-/-}$ hippocampal neurons were treated with Tm (1 μg/ml) together with different concentrations of salubrinal (5-75 μM). Cell death was evaluated by immunocytochemical staining for cleaved caspase-3. n=3 experiments. Data are shown as mean ± SEM. *p < 0.05, ***p< 0.001 between two genotypes and # p < 0.05, ## p < 0.01, ### p<0.001 compared to normal conditions by a two-way ANOVA followed by the Bonferroni test. Scale bar: 20 μm.

Figure S8. Neuroprotection by ATF6β in KA-injected mice and expression of *Calr* in *Calr*$^{+/-}$ mice. A, Brain sections containing the CA3 area of the hippocampus from WT and *Atf6b*$^{-/-}$ mice were subjected to Nissl staining. The right graph depicts the number of surviving CA3 neurons. n=6-8 mice. Data are shown as mean ± SEM. **p < 0.01, ***p < 0.001 by a two-way ANOVA followed by the Bonferroni test. Scale bar: 100 μm. B, Total RNA was isolated from WT and *Calr*$^{+/-}$ brains including the CA3 region of the hippocampus and qRT-PCR analysis of *Calr* and *Canx* was performed. n=4 mice. Data are shown as mean ± SEM. *p < 0.001 by the Mann Whitney-U test. C, Protein extracts from WT and *Calr*$^{+/-}$ brains

including the CA3 region of the hippocampus were subjected to western blotting for the indicated proteins.

Figure S9. Neuronal activity and death after KA injection into the hippocampi of WT and *Atf6b*$^{-/-}$ mice. A, Total RNA was isolated from the CA3 area of the hippocampus from WT and *Atf6b*$^{-/-}$ mice after KA injection. n=3–5 mice. Data are shown as mean ± SEM. *p < 0.01, **p < 0.01 between two genotypes by a two-way ANOVA followed by the Bonferroni test. B, Brain sections including the CA3 area of the hippocampus obtained from WT and *Atf6b*$^{-/-}$ mice at 1 h after KA injection were subjected to immunohistochemical staining for c-Fos. The right graph depicts the number of c-Fos-positive neurons. n=3 mice. Data are shown as mean ± SEM. *p < 0.05 by a two-way ANOVA followed by the Bonferroni test. C, D, Brain sections including the CA3 area of the hippocampus obtained from WT and *Atf6b*$^{-/-}$ mice 3 days after injection with 2-APB or salubrinal alone (n=4 mice) (C) or with KA plus 2-APB and KA plus salubrinal (n=6 mice) (D) were subjected to Nissl staining. The right graph depicts the number of surviving CA3 neurons. Data are shown as mean ± SEM. **p < 0.01, ***p < 0.001 by a two-way ANOVA followed by the Bonferroni test.

Figure S10. Spatial memory tested by using the Morris water maze. A, The experimental protocol for the Morris water maze. B, Averaged escape latencies are plotted (WT, black solid line; *Atf6b*$^{-/-}$, black dotted line). WT, n = 11; *Atf6b*$^{-/-}$, n = 7. Data are shown as mean ± SEM. C and D, The time spent in the approach zone (C) and evacuation zone (D) was measured on

day 5 with the target removed after the final goal-seeking session. WT, n = 11; *Atf6b*$^{-/-}$, n = 7. Data are shown as mean ± SEM.

Figure S11. Original images of western blots from Figures 1E, 2B, and 3B. Western blots of ATF6β from Figure 1E (A), Calreticulin, Calnexin, GRP78, and GAPDH from Figure 2B (B), and Calreticulin, Calnexin, GRP94, GRP78, and GAPDH from Figure 3B (C). Membranes were cut prior to incubation with antibodies. Areas of interest are marked with pink rectangles.

Figure S12. Uncropped images of western blotting from Figures 5A, 6B, and S8 C. Western blots of Calreticulin, Canexin, and GAPDH from Figure 5A (A), Calreticulin, Calnexin, GRP94, GRP78, and GAPDH from Figure 6B (B), and Calreticulin, Calnexin, GAPDH from Figure S8 C (C). Membranes were cut prior to incubation with antibodies. Areas of interest are marked with pink rectangles.

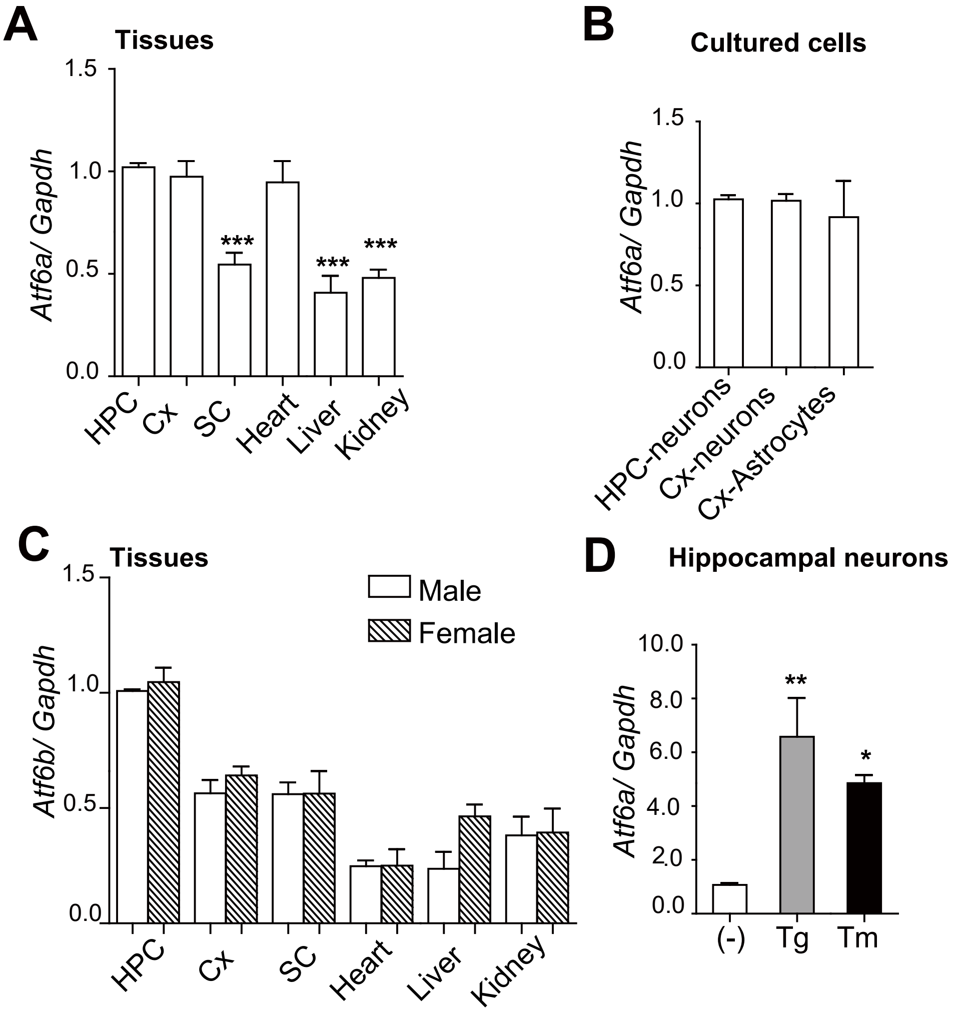
A
Tissues
Atf6a/ Gapdh
1.5
1.0
0.5
0.0
***
***
***
HPC
Cx
SC
Heart
Liver
Kidney
B
Cultured cells
Atf6a/ Gapdh
1.5
1.0
0.5
0.0
HPC-neurons
Cx-neurons
Cx-Astrocytes
C
Tissues
Male
Female
Atf6b/ Gapdh
1.5
1.0
0.5
0.0
HPC
Cx
SC
Heart
Liver
Kidney
D
Hippocampal neurons
Atf6a/ Gapdh
10.0
8.0
6.0
4.0
2.0
0.0
**
*
(-)
Tg
Tm

Figure S1

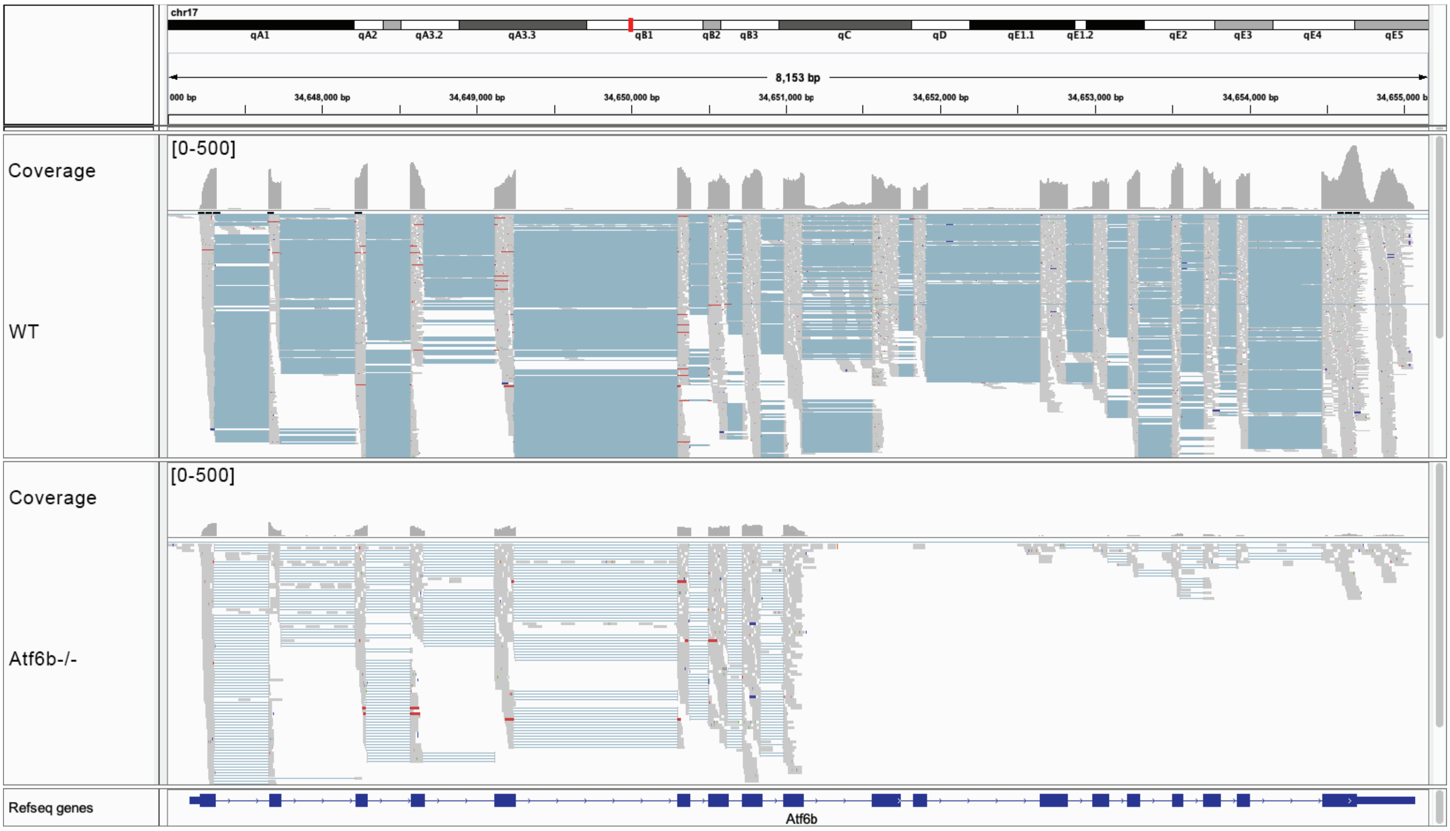

chr17
qA1
qA2
qA3.2
qA3.3
qB1
qB2
qB3
qC
qD
qE1.1
qE1.2
qE2
qE3
qE4
qE5
8,153 bp
000 bp
34,648,000 bp
34,649,000 bp
34,650,000 bp
34,651,000 bp
34,652,000 bp
34,653,000 bp
34,654,000 bp
34,655,000 b
Coverage
[0-500]
WT
Coverage
[0-500]
Atf6b-/-
Refseq genes
Atf6b

Figure S2

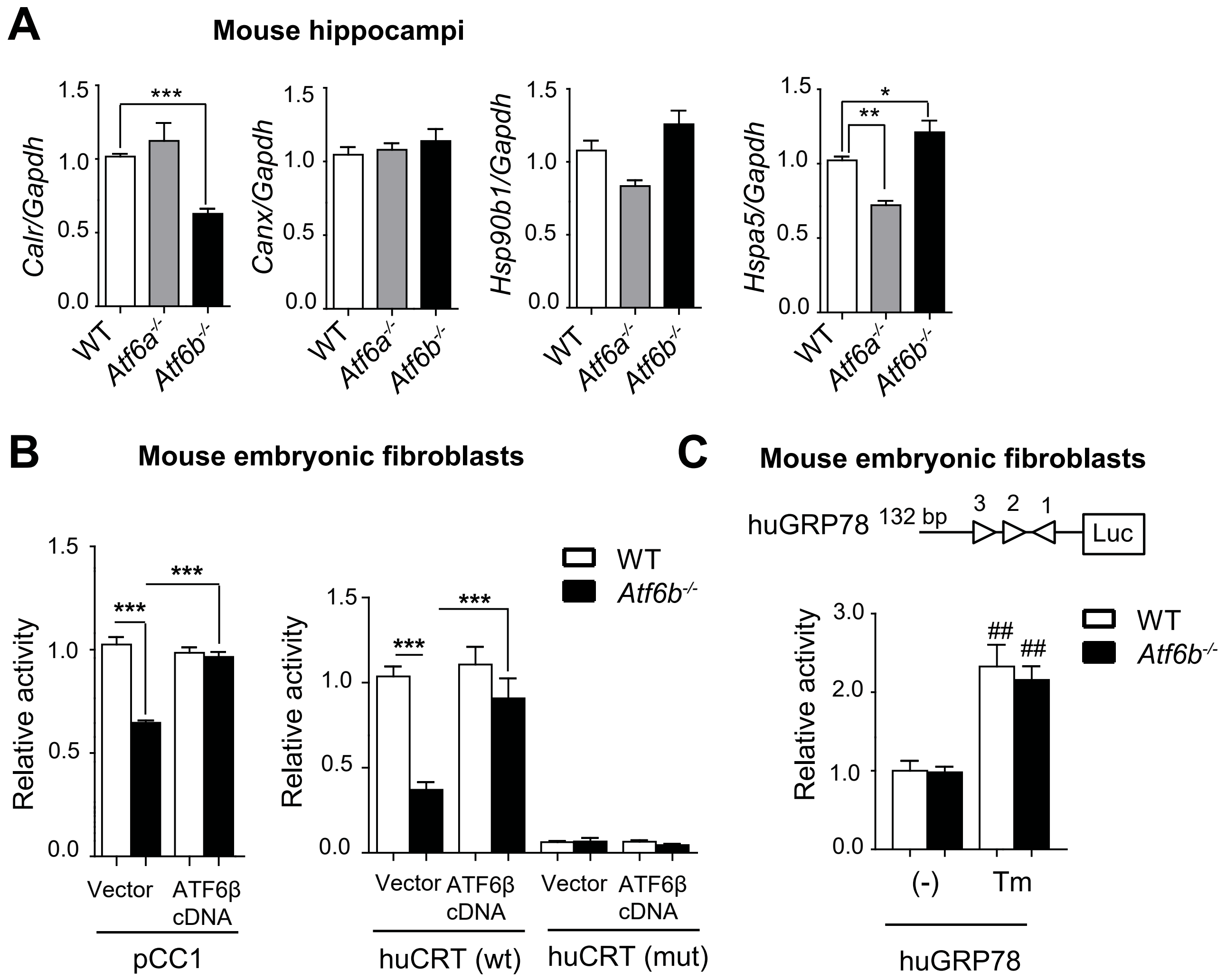
A
Mouse hippocampi
Calr/Gapdh
Canx/Gapdh
Hsp90b1/Gapdh
Hspa5/Gapdh
1.5
1.0
0.5
0.0
***
**
*
WT
Atf6a-/-
Atf6b-/-
B
Mouse embryonic fibroblasts
Relative activity
Vector
ATF6β cDNA
pCC1
huCRT (wt)
huCRT (mut)
WT
Atf6b-/-
C
Mouse embryonic fibroblasts
huGRP78
132 bp
3 2 1
Luc
3.0
2.0
##
(-)
Tm
huGRP78

Figure S3

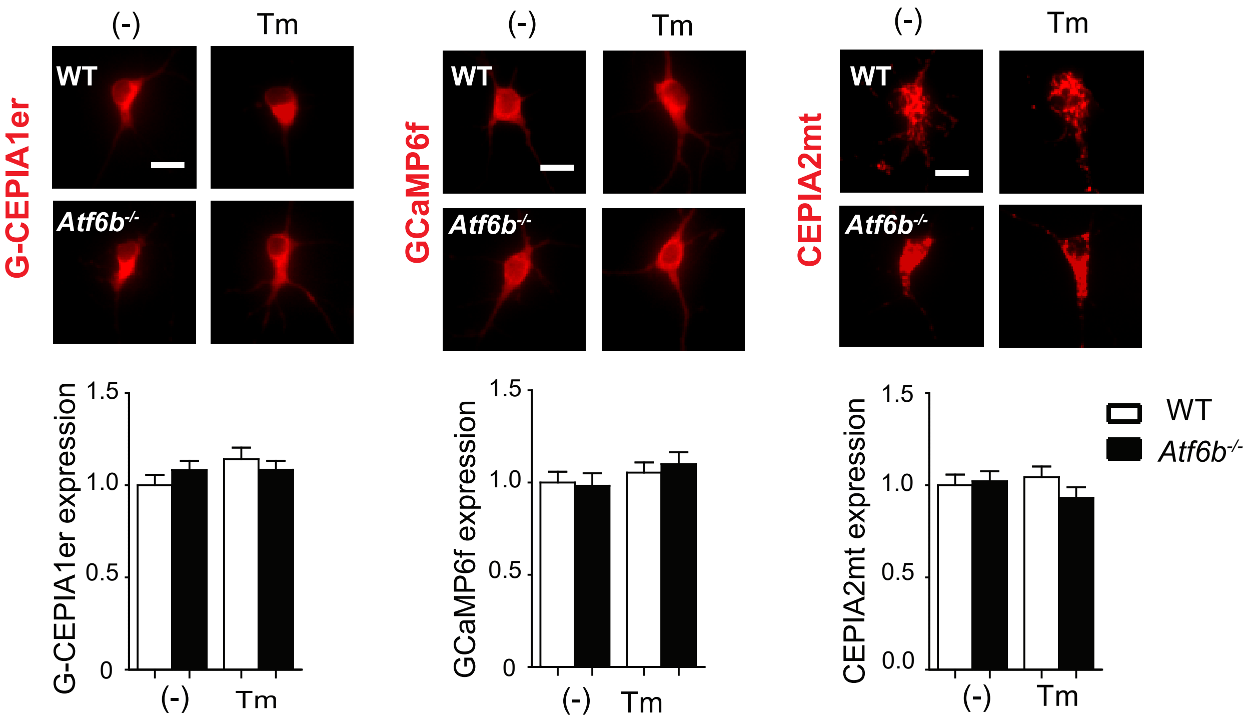
(-)
Tm
G-CEPIA1er
GCaMP6f
CEPIA2mt
WT
Atf6b-/-
G-CEPIA1er expression
GCaMP6f expression
CEPIA2mt expression
1.5
1.0
0.5
0
0.0
WT
Atf6b-/-

Figure S4

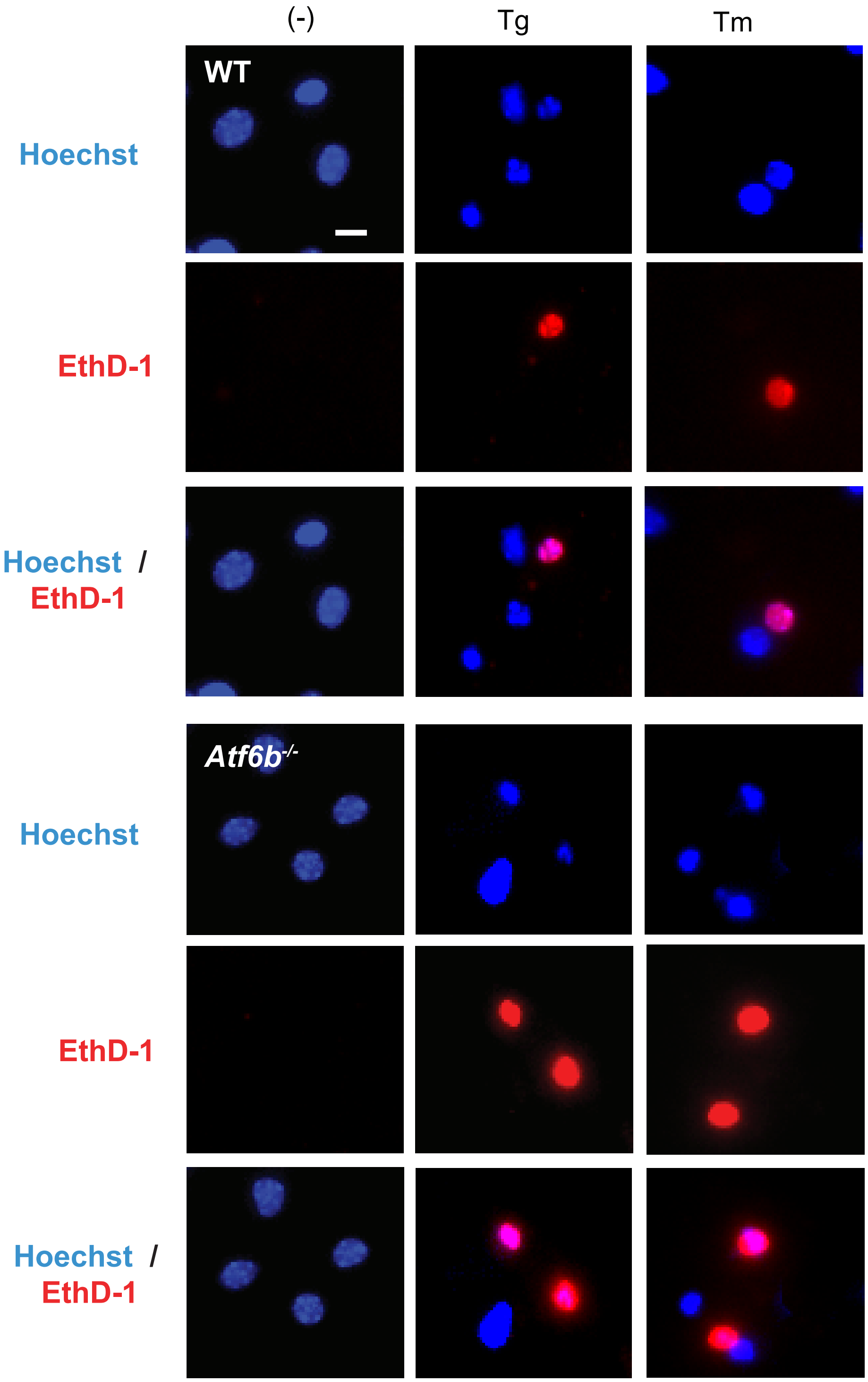
(-)
Tg
Tm
WT
Hoechst
EthD-1
Hoechst / EthD-1
Atf6b-/-
Hoechst
EthD-1
Hoechst / EthD-1

Figure S5

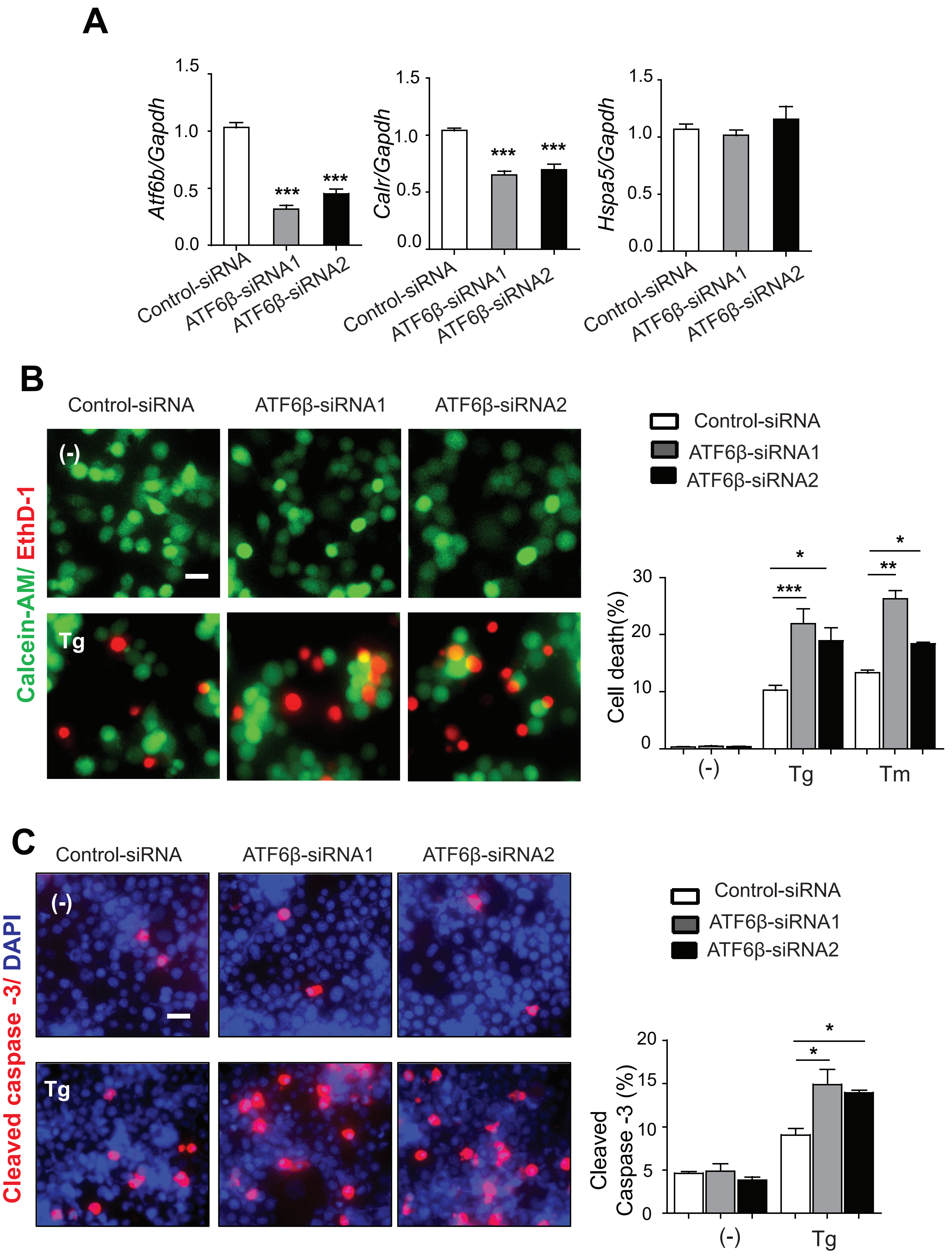

A
Atf6b/Gapdh
Calr/Gapdh
Hspa5/Gapdh
1.5
1.0
0.5
0.0
***
Control-siRNA
ATF6β-siRNA1
ATF6β-siRNA2
B
Control-siRNA
ATF6β-siRNA1
ATF6β-siRNA2
Calcein-AM/ EthD-1
(-)
Tg
Cell death(%)
30
20
10
0
(-)
Tg
Tm
C
Control-siRNA
ATF6β-siRNA1
ATF6β-siRNA2
Cleaved caspase -3/ DAPI
(-)
Tg
Cleaved Caspase -3 (%)
20
15
10
5
0

Figure S6

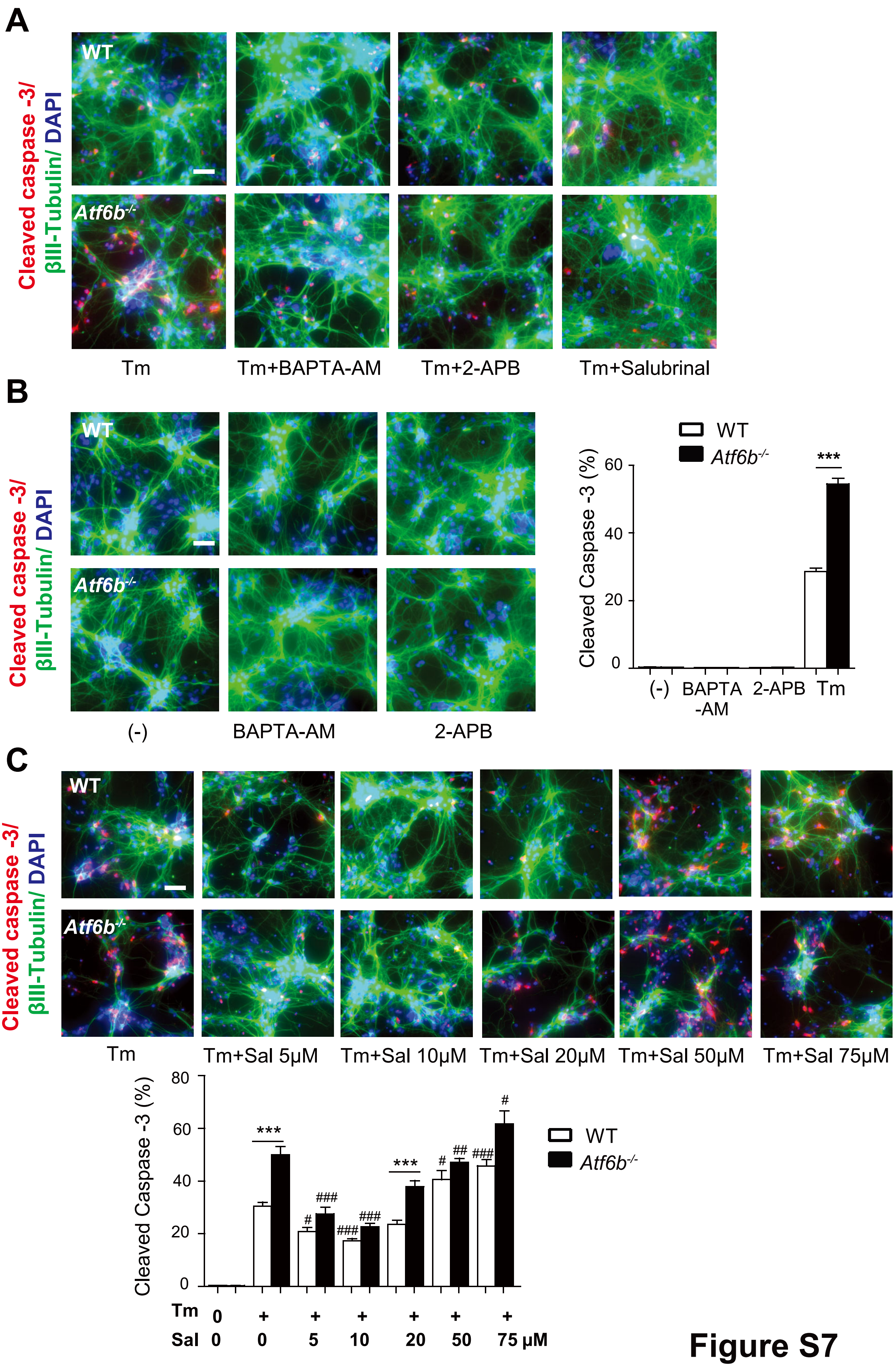
A
WT
Atf6b-/-
Cleaved caspase -3/ βIII-Tubulin/ DAPI
Tm
Tm+BAPTA-AM
Tm+2-APB
Tm+Salubrinal
B
WT
Atf6b-/-
Cleaved caspase -3/ βIII-Tubulin/ DAPI
(-)
BAPTA-AM
2-APB
WT
Atf6b-/-
Cleaved Caspase -3 (%)
***
(-)
BAPTA -AM
2-APB
Tm
C
WT
Atf6b-/-
Cleaved caspase -3/ βIII-Tubulin/ DAPI
Tm
Tm+Sal 5μM
Tm+Sal 10μM
Tm+Sal 20μM
Tm+Sal 50μM
Tm+Sal 75μM
Cleaved Caspase -3 (%)
***
###
#
###
###
***
#
##
###
#
WT
Atf6b-/-
Tm 0 + + + + + +
Sal 0 0 5 10 20 50 75 μM

Figure S7

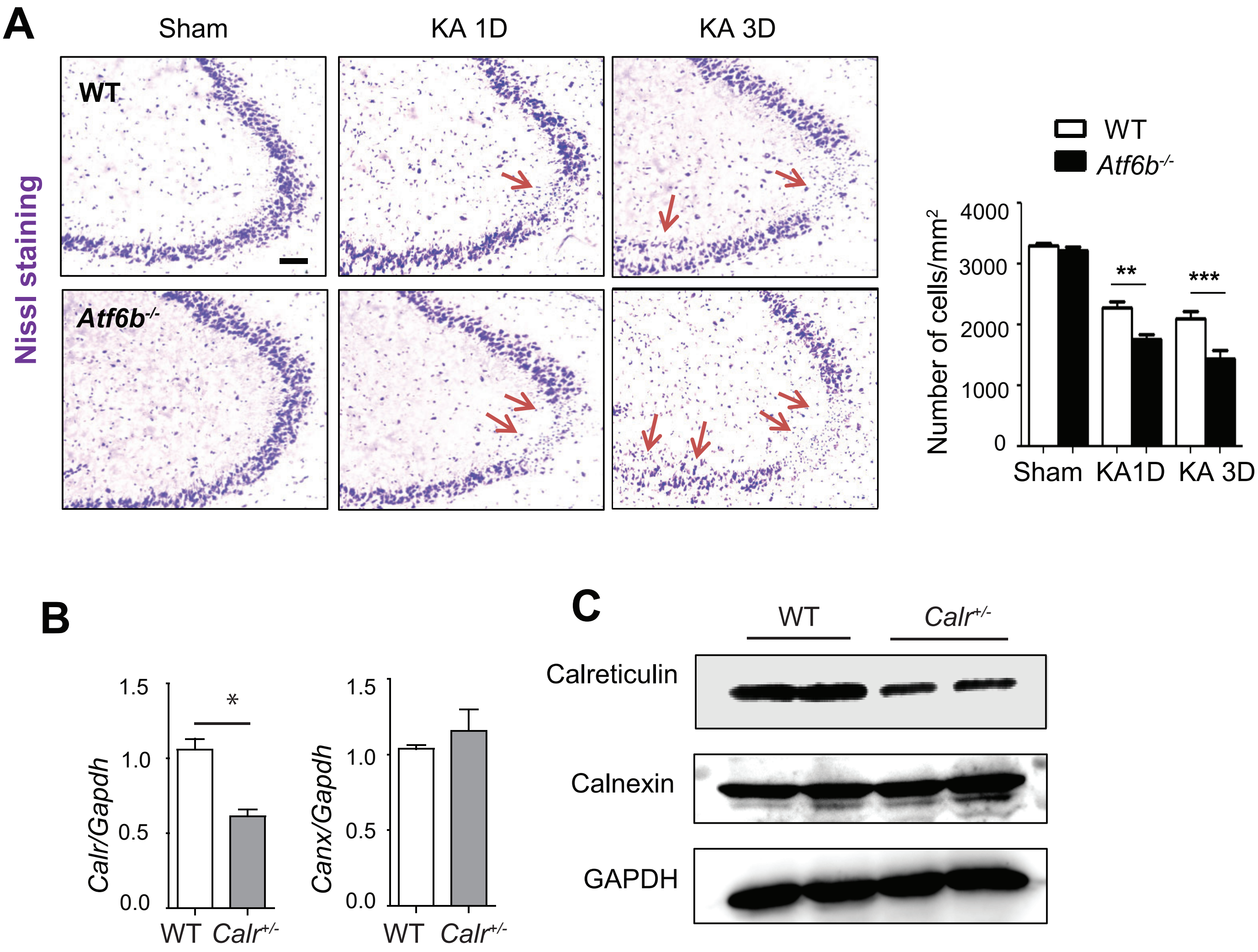
A
Sham
KA 1D
KA 3D
Nissl staining
WT
Atf6b-/-
WT
Atf6b-/-
Number of cells/mm2
4000
3000
2000
1000
0
**
***
Sham
KA1D
KA 3D
B
Calr/Gapdh
1.5
1.0
0.5
0.0
*
WT
Calr+/-
Canx/Gapdh
1.5
1.0
0.5
0.0
WT
Calr+/-
C
WT
Calr+/-
Calreticulin
Calnexin
GAPDH

Figure S8

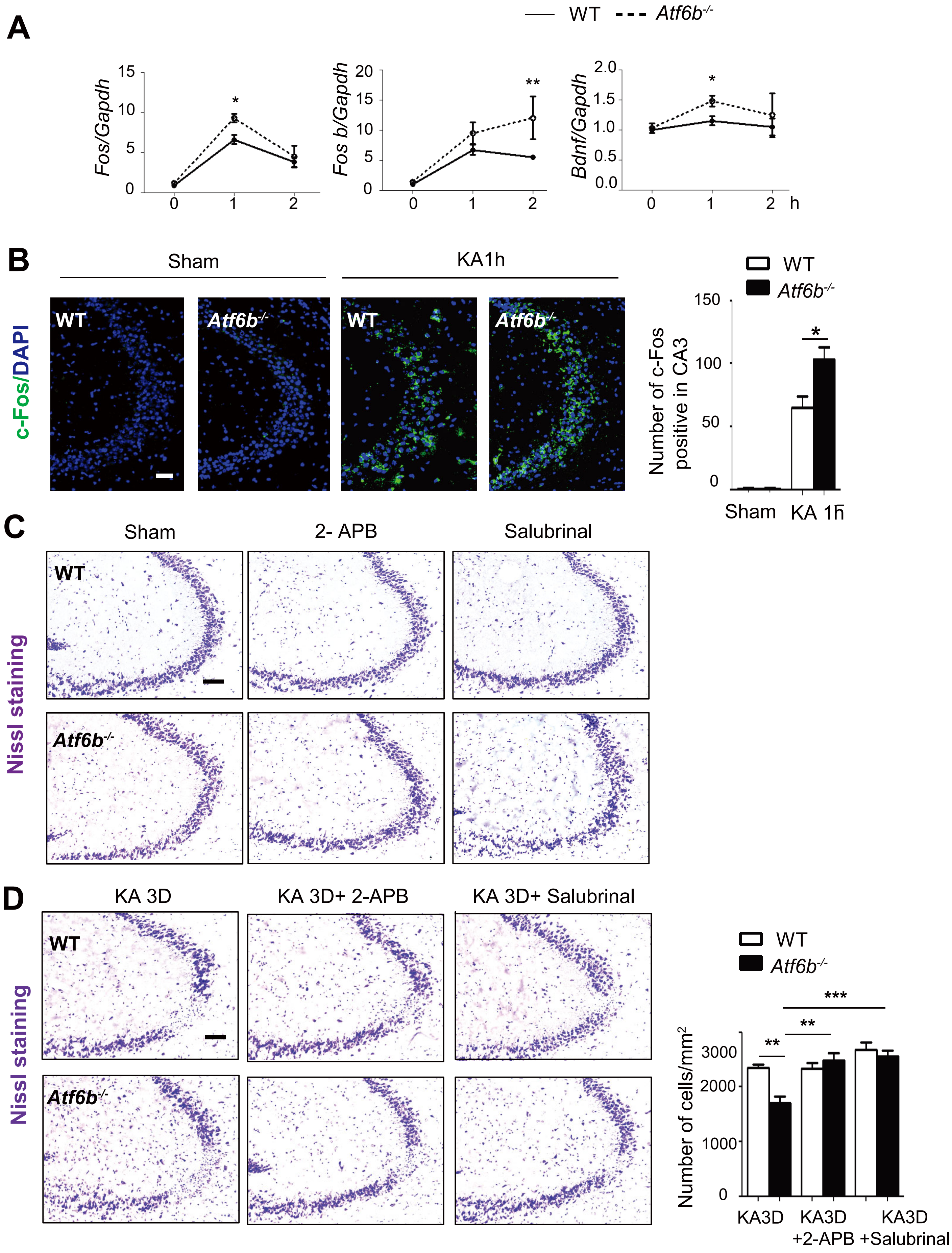
A
WT
Atf6b-/-
Fos/Gapdh
Fos b/Gapdh
Bdnf/Gapdh
h
B
Sham
KA1h
c-Fos/DAPI
WT
Atf6b-/-
Number of c-Fos positive in CA3
Sham
KA 1h
C
Sham
2- APB
Salubrinal
Nissl staining
D
KA 3D
KA 3D+ 2-APB
KA 3D+ Salubrinal
Number of cells/mm2
KA3D
KA3D +2-APB
KA3D +Salubrinal

Figure S9

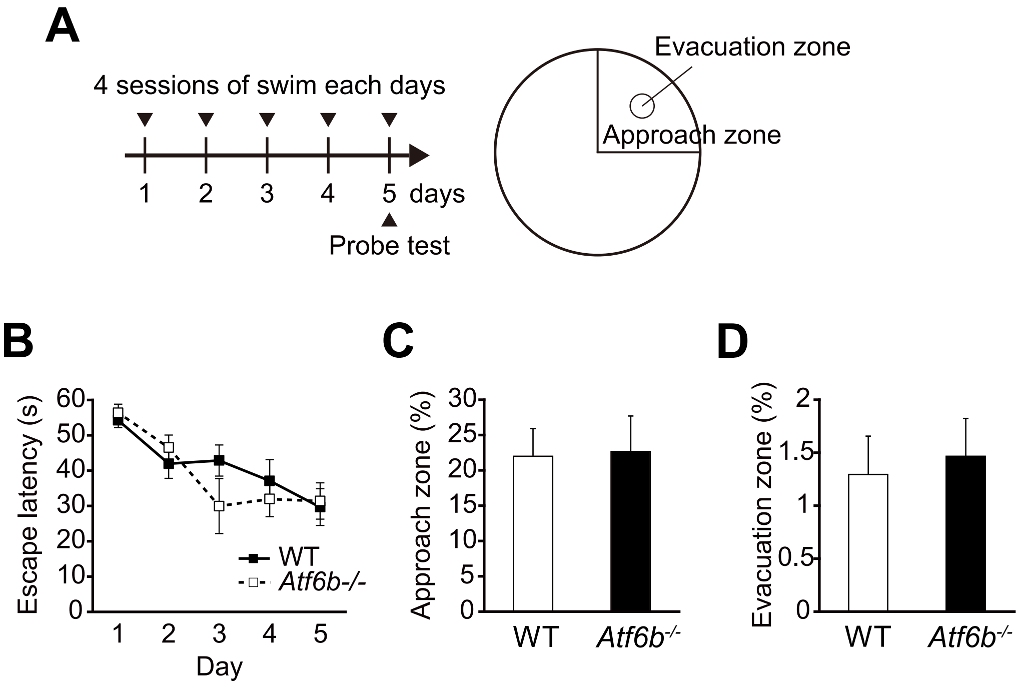
A
4 sessions of swim each days
1
2
3
4
5
days
Probe test
Evacuation zone
Approach zone
B
Escape latency (s)
60
50
40
30
20
10
0
1
2
3
4
5
Day
WT
Atf6b-/-
C
Approach zone (%)
30
25
20
15
10
5
0
WT
Atf6b-/-
D
Evacuation zone (%)
2
1.5
1
0.5
0
WT
Atf6b-/-

Figure S10

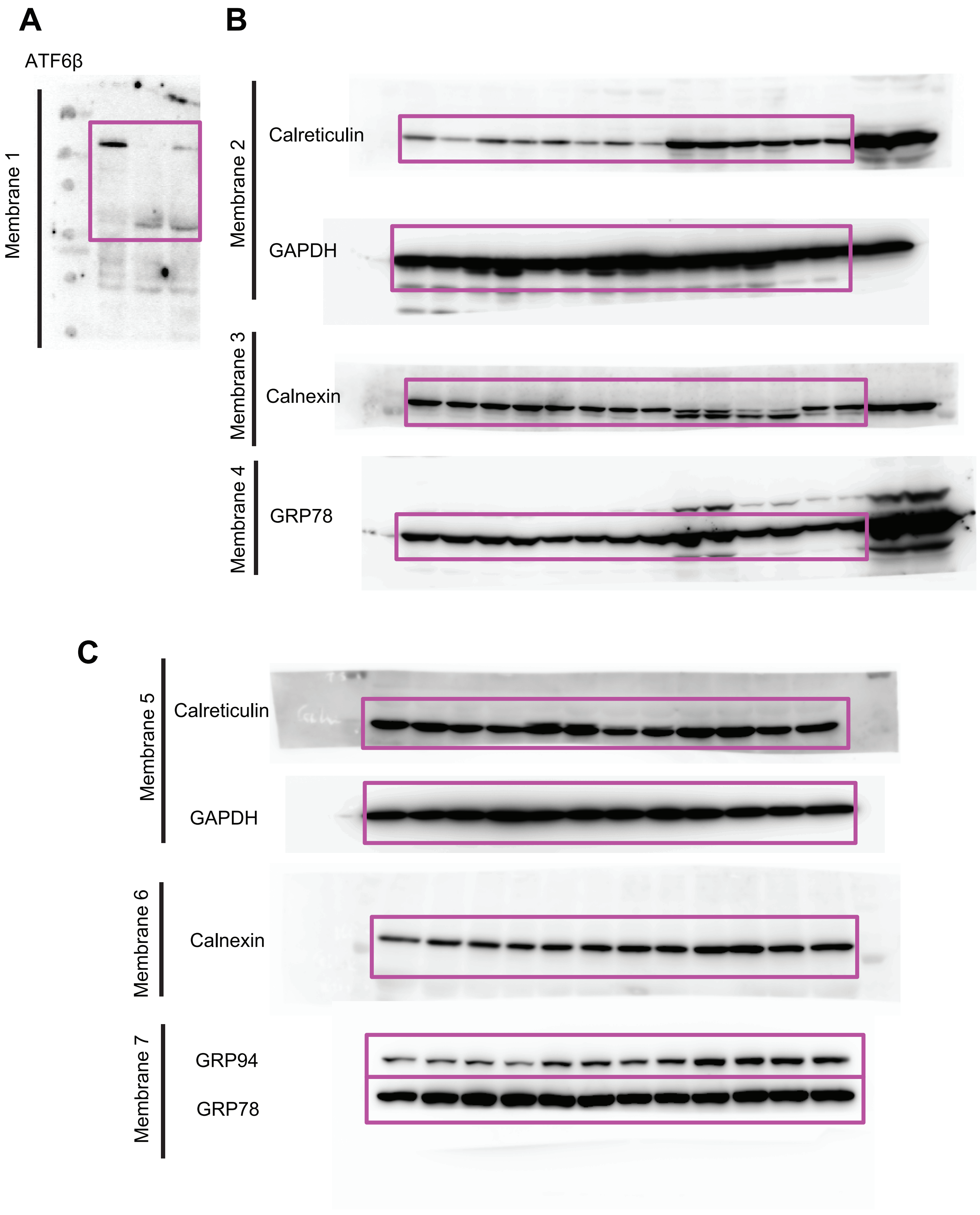
A
ATF6β
Membrane 1
B
Membrane 2
Calreticulin
GAPDH
Membrane 3
Calnexin
Membrane 4
GRP78
C
Membrane 5
Calreticulin
GAPDH
Membrane 6
Calnexin
Membrane 7
GRP94
GRP78

Figure S11

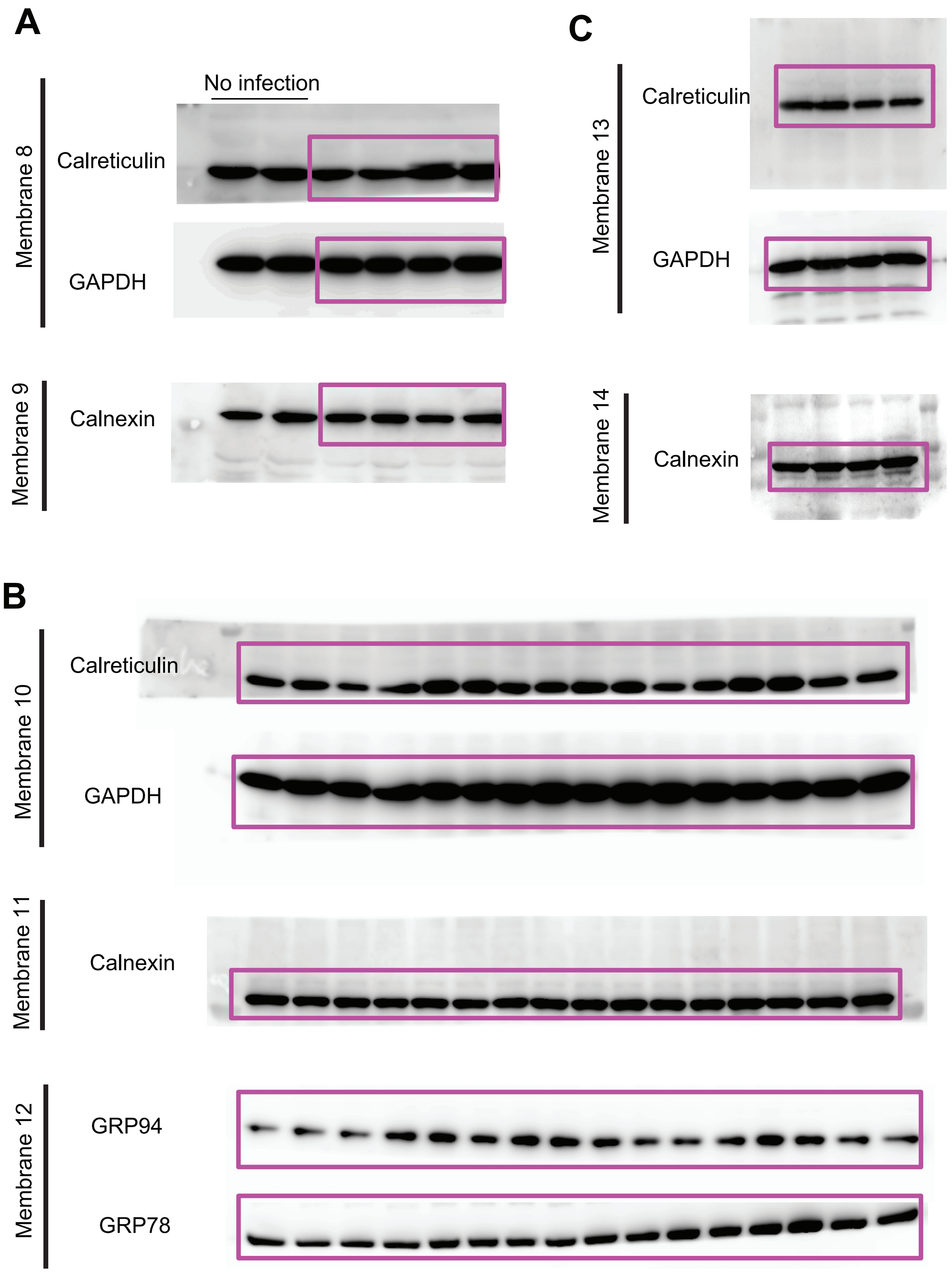

A
No infection
Membrane 8
Calreticulin
GAPDH
Membrane 9
Calnexin
C
Membrane 13
Calreticulin
GAPDH
Membrane 14
Calnexin
B
Membrane 10
Calreticulin
GAPDH
Membrane 11
Calnexin
Membrane 12
GRP94
GRP78

Figure S12
